# Folate supplementation as a beneficial add‐on treatment in relieving depressive symptoms: A meta‐analysis of meta‐analyses

**DOI:** 10.1002/fsn3.4073

**Published:** 2024-03-08

**Authors:** Shan Gao, Awais Khalid, Ehsan Amini‐Salehi, Nima Radkhah, Parsa Jamilian, Mohaddeseh Badpeyma, Meysam Zarezadeh

**Affiliations:** ^1^ Clinical Nutrition Department Xianyang Central Hospital Xianyang City Shaanxi Province China; ^2^ Department of Physics, College of Science and Humanities in Al‐Kharj Prince Sattam bin Abdulaziz University Al‐Kharj Saudi Arabia; ^3^ School of Medicine Guilan University of Medical Sciences Rasht Iran; ^4^ School of Nutrition and Food Sciences Tabriz University of Medical Sciences Tabriz Iran; ^5^ Keele Medical School Keele University Staffordshire UK; ^6^ Student Research Committee Tabriz University of Medical Sciences Tabriz Iran

**Keywords:** depression, folate, supplementation, umbrella meta‐analysis

## Abstract

The results of meta‐analyses investigating the role of folate on depression are conflicting. The aim of this umbrella meta‐analysis was to obtain an overall effect and give a concise and resolving conclusion. International scientific databases including PubMed, Scopus, and Web of Science were searched up to Oct 2023. All observational and interventional meta‐analyses investigating the role of folate in depression were included in the study. Random‐effects model was employed to obtain pooled results. *I* ^2^ statistics and Cochrane *Q* test were used to assess the between‐study heterogeneity. The quality of included meta‐analyses was evaluated using the Assessing the Methodological Quality of Systematic Reviews 2 (AMSTAR2) questionnaire. Overall 11 studies were included, of which 8 studies went under quantitative evaluation. The results indicated that folate supplementation significantly relieved depression symptoms [(SMD: −0.42; 95% CI: −0.57, −0.27, *p* < .001; *I* ^2^ = 0.0%, p‐heterogeneity = 0.554) (WMD: −3.20; 95% CI: −4.00, −2.41, *p* < .001, *I* ^2^ = 14.8%, p‐heterogeneity = 0.318)] with low levels of heterogeneity. Also, based on observational studies, folate insufficiency significantly increased the odds ratio of depression by 35% (OR:1.35; 95% CI: 1.27, 1.42, *p* < .001, *I* ^2^ = 8.7%, *p*‐heterogeneity = 0.350). The findings support the fact that folate supplementation could be suggested as an efficacious and adjuvant agent in the alleviation of depression symptoms along with routine medications.

## Graphical abstract

Folate supplementation significantly decreased symptoms of depression. The decreasing effect is more robust when supplementation lasts for >10 weeks. Low serum level of folate is significantly associated with odds of depression.

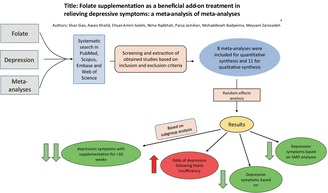

## 1 INTRODUCTION

Depression is recognized as one of the most common mental disorders that has substantial impacts on public health (Bender et al., 2017; Musazadeh et al., 2023). Based on the report of the World Health Organization, over 264 million people suffer from depressive disorders worldwide (James et al., 2018). It is related to significant complications like changes in mood, appetite, and weight, loss of interest, lack of energy, anhedonia, and feelings of worthlessness. Untreated depression will result in increase in morbidity and mortality (Altaf et al., 2021; Smith et al., 2021). Although the disease burden and mortality associated with depressive disorders are substantial, current medications and psychotherapies are not beneficial to almost 60% of patients with depressive disorders (Bender et al., 2017; Strawbridge et al., 2017). Additionally, antidepressants could have adverse effects and, as a result, lead to refusal to continuing the treatment process (Davies & Read, 2019; Lincoln et al., 2016). Thus, it would appear that current treatments for depression might not be completely effective for some individuals, and it becomes imperative to provide complementary treatments for it.

**FIGURE 1:**
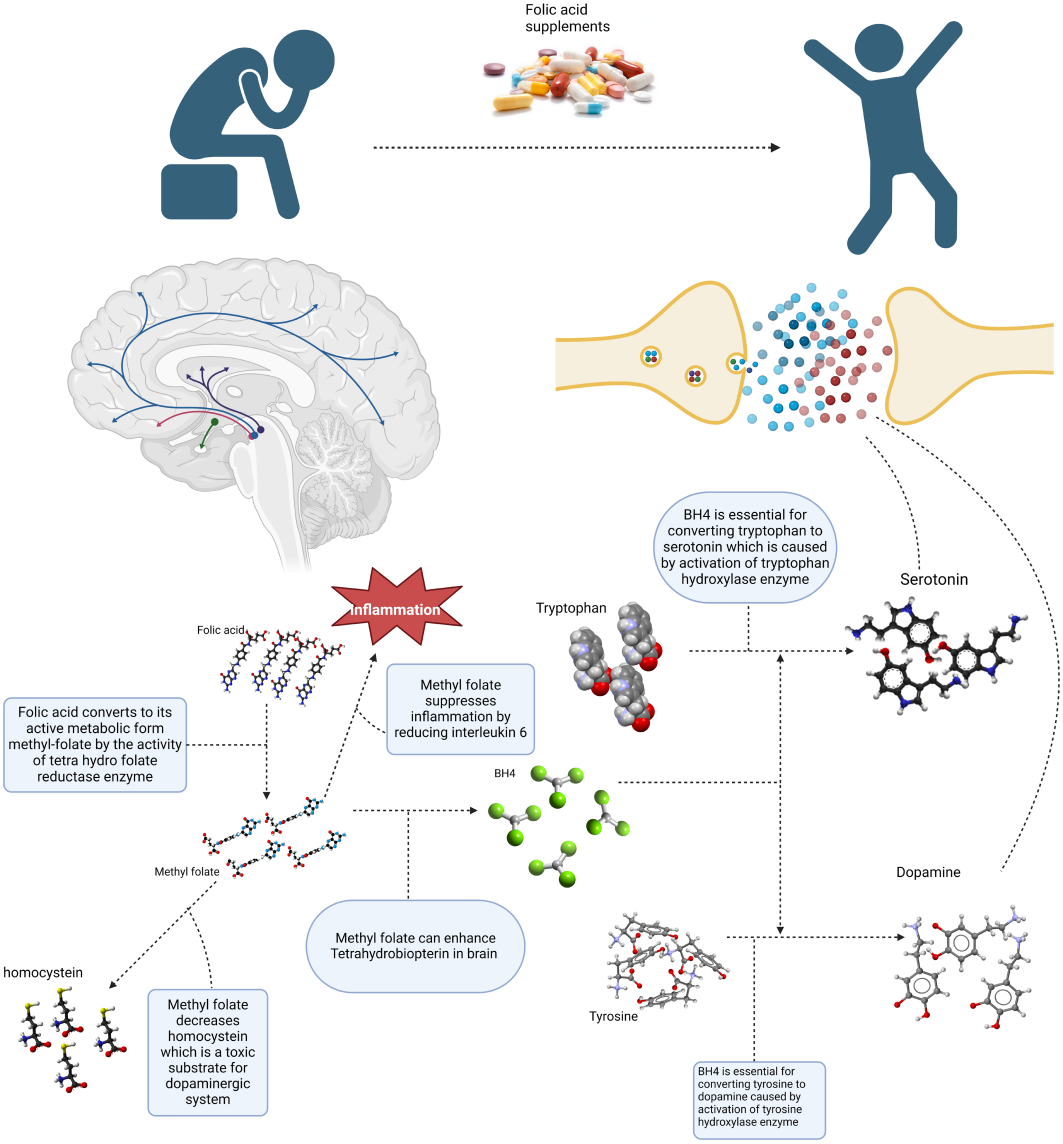
Mechanisms of action of folate on relieving depression symptoms.

It has been shown that there is an association between dietary intake and depression (Yu et al., 2023). Folate or vitamin B9 is one of the water‐soluble vitamins that may play a role in depression (Lyon et al., 2020). Folate plays a fundamental role in maintaining one‐carbon metabolism for cell division and nucleic acid synthesis, regulation of gene expression, interconversion of amino acids and neurotransmitter synthesis, and neurological development during pregnancy and infancy (Gianfredi et al., 2022; Himayda et al., 2018; Hoepner et al., 2021; Jin et al., 2022; Xie et al., 2019; Yang et al., 2021). Growing research demonstrated that depression might be linked to folate deficiency. Particularly, studies indicate a link between folate deficiency and an increased risk of depression, more severe depressive symptoms, more prolonged episodes of depression, increased risk of depressive symptom relapse, and poor response to antidepressants (Bender et al., 2017; Liwinski & Lang, 2023; Martínez‐Cengotitabengoa & González‐Pinto, 2017). However, some studies also showed that low‐serum folate levels have been positively associated with depression (Huang et al., 2018; Kaner et al., 2015; Khalili et al., 2022; Lin et al., 2020; Seppälä et al., 2013), whereas others have found no difference (Gargari et al., 2012; Gilbody et al., 2007; Kendrick et al., 2008). As a result, a substantial body of evidence suggests the introduction of supplemental folate in preventing and treating depression at the population and individual levels (Himayda et al., 2018). Figure 1 shows how folate could be related to depression.

The aim of the current meta‐analysis of meta‐analyses was to determine the effectiveness of folate supplementation or association of higher serum levels of folate on/with depressive disorders to provide insight into the subject and resolve controversies.

## 2 METHODS

This study was conducted according to the guiding principle of the Preferred Reporting Items for Systematic Reviews and Meta‐analysis (PRISMA) (Moher et al., 2015). The protocol of the study was registered on PROSPERO (Code: CRD42023403671).

### 2.1 Search strategy and study selection

Scopus, PubMed, Web of Science databases, and Google Scholar were systematically searched up to the end of October 2023 by using the below pattern; (“((((((folate[Title/Abstract]) OR (“folic acid”[Title/Abstract])) OR (tetrahydrofolate[Title/Abstract])) OR (“vitamin B9”[Title/Abstract])) OR (“Folic Acid”[Mesh])) AND ((((((depression[Title/Abstract]) OR (depressive[Title/Abstract])) OR (“depressive disorder”[Title/Abstract])) OR (“major depressive disorder”[Title/Abstract])) OR (depress*[Title/Abstract])) OR (“Depression”[Mesh] OR “Depressive Disorder”[Mesh] OR “Depression, Postpartum”[Mesh] OR “Depressive Disorder, Major”[Mesh]))) AND (((meta‐analysis [Publication Type]) OR (meta‐analysis[Title/Abstract])) OR (meta[Title/Abstract]))”). The data extraction process was started on March 25, 2023. Also, the wild‐card term “*” was used to enhance the search sensitivity. Only articles in the English language were considered for eligibility.

### 2.2 Inclusion and exclusion criteria

The inclusion criteria were defined using the following PICO: population (adults of >18 years of age); intervention/exposure (I: folate supplementation or insufficiency); comparison (C: control or placebo group); and outcome (O: depression symptoms). Interventional and observational meta‐analysis studies investigating the effects/association of folate on/with depression symptoms reporting the effect sizes (ESs) and corresponding confidence intervals (CIs) were considered eligible for inclusion. All studies with designs other than “meta‐analysis” were excluded.

### 2.3 Methodological quality assessment and grading of the evidence

In order to assess the reliability and quality of the included meta‐analyses, Assessing the Methodological Quality of Systematic Reviews 2 (AMSTAR2) questionnaire was employed by two independent researchers (PJ and NR) (Shea et al., 2017). It is composed of 16 items scored which should be answered with “Yes,” “Partial Yes,” “No,” or “Not a Meta‐analysis.” There are four levels of quality for the AMSTAR2 checklist: “Critically low quality,” “low quality,” “moderate quality,” and “high quality.”

### 2.4 Study selection and data extraction

The primary screening (i.e., screening of the titles and abstracts) according to the eligibility criteria was performed by two reviewers (NR and PJ). The full texts of potentially eligible articles were obtained, and two reviewers (NR, PJ) independently assessed each reference's final eligibility. Any disagreements were resolved through discussion with a third reviewer (MZ). The following data were extracted from the eligible articles: publication year, sample size, study location and duration folate supplementation, ES [including weighted mean difference (WMD), standardized mean difference (SMD), and odds ratio (OR)], and CIs for depression symptoms.

### 2.5 Data synthesis and statistical approach

Random‐effects model was employed to estimate the overall ES, employing restricted maximum‐likelihood method (REML) (Higgins et al., 2019). The *I* ^2^ statistic and Cochrane's *Q*‐test were utilized to detect heterogeneity. *I* ^2^ value above 50% or *p*‐value less than .1 for the *Q*‐test was regarded as considerable between‐study heterogeneity (Higgins et al., 2019). Due to the natural variances, the analysis was carried out independently for SMD and WMD. Also, the observational meta‐analyses were analyzed separately. In order to identify the potential sources of heterogeneity and report ES across various independent variables, subgroup analyses were conducted based on predefined variables including duration of intervention, target sample, participants' mean age, and supplementation dosage. Sensitivity analysis was performed to determine the effect of one specific study removal on the overall effect size. Formal Egger et al. (1997) and Begg and Mazumdar (1994) tests, as well as visual examination of the funnel plots (Doleman et al., 2020), were employed to address publication bias for outcomes with at least 10 observations. The overall results were corrected using the “trim and fill” method, where asymmetric distribution of the studies was observed and suspected to be due to publication bias. All statistical analyses were performed using STATA version 16.0 (Stata Corporation, College Station, TX, US). Unless otherwise stated, a P‐value of less than 0.05 was considered significant.

## 3 RESULTS

### 3.1 Study selection

**FIGURE 2:**
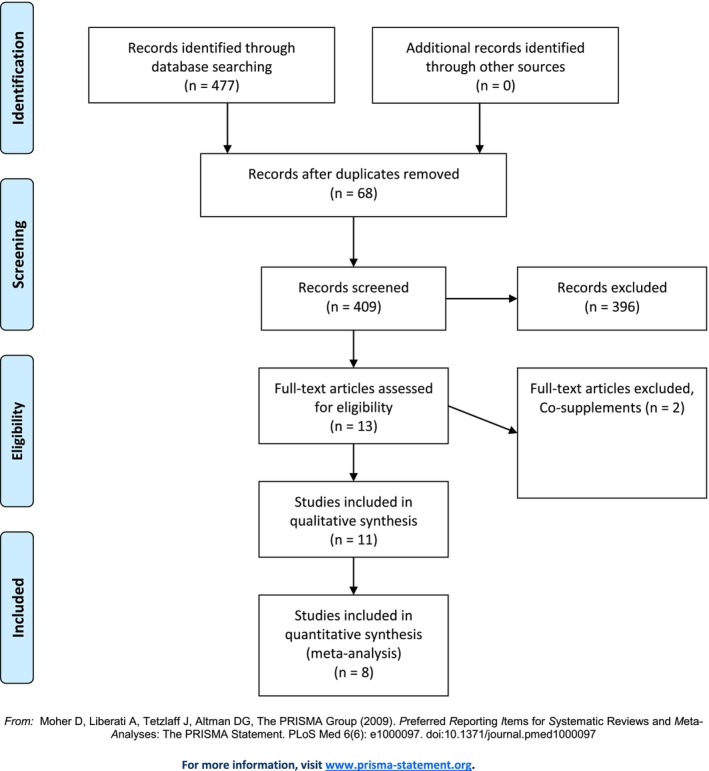
The process study selection shown on PRISMA flow chart.

Overall 477 studies were retrieved from searching databases among which 68 studies were duplicates. After screening remaining 409 articles by titles and abstracts, 13 articles went under careful evaluation by full‐text version among which 8 articles (Al Maruf et al., 2022; Altaf et al., 2021; Gilbody et al., 2007; Jin et al., 2022; Khalili et al., 2022; Petridou et al., 2016; Roberts et al., 2018; Taylor et al., 2004) with 11 datasets fully met the inclusion criteria for quantitative synthesis. Considering the qualitative synthesis, 11 studies were included in total. The process of study selection is demonstrated in Figure 2.

### 3.2 Study characteristics

In the present study, three meta‐analyses with 1995 participants from 14 RCTs evaluating the effect of folate supplementation on depressive symptoms which reported their results based on SMD analysis were included (Al Maruf et al., 2022; Altaf et al., 2021; Roberts et al., 2018). The eligible studies were conducted between 2018 and 2021. The participants' average age ranged between 40 and 44 years. The duration of the intervention was between 6 and 11 weeks. Supplementation varied between 500 μg folic acid and 15 mg methyltetrahydrofolate per day. All included RCTs used folate as an adjuvant to other standard and routine psychiatric drug therapies.

Moreover, three meta‐analyses of 1046 participants from 14 RCTs that reported WMD were included (Altaf et al., 2021; Khalili et al., 2022; Taylor et al., 2004). The included studies were conducted between 2004 and 2022. The participants' mean age was between 31 and 43 years. The intervention duration was between 5 and 16 weeks. Supplementation dosage varied between 500 μg folic acid and 15 mg methyltetrahydrofolate per day. All included RCTs used folate as an adjuvant to other standard and routine psychiatric drug therapies. The characteristics of interventional meta‐analyses are demonstrated in Table 1.

**TABLE 1:** Characteristics of meta‐analyses of interventional studies investigating the effects of folate supplementation on depression disorder.

Study, year, country	Participants	Study number	Gender	Mean age	Therapy	Supplements	Mean duration	Outcome	Quality
Taylor et al. (2004) UK	*1‐MDD RBC folate <200 μg/L *2‐MDD	2	F/M	43.9	*1‐Fluoxetine *2‐standard psychotropic treatments	*1–0.5 mg folic acid *2–15 mg methyltetrahydrofolate	15 week	Depression ↓	Yes (randomization, concealment of allocation, and blinding) 2/2 Moderate
Khalili et al. (2022) Iran	MDD	5	F/M	35.41	*1–20 mg citalopram *2–20 mg fluoxetine *3–20 mg fluoxetine *4–20 mg fluoxetine *5‐sertraline	*1–2.5 mg folic acid *2–10 mg folic acid *3–0.5 mg folic acid *4–1.5 mg folic acid *5–2 mg folic acid	15 week	Depression ↓	Yes (Cochrane) 2/5 High
Khalili et al. (2022) Iran	Eating disorders and MDD	3	F/M	31.12	*1–20 mg citalopram *2‐NR *3–20 mg fluoxetine	*1–2.5 mg folic acid *2–5 mg folic acid *3–1.5 mg folic acid	15 week	Depression ↓	Yes (Cochrane) 2/3 High
Altaf et al. (2021) US (Reporting WMD)	NR	4	F/M	41.4	*1‐Fluoxetine (20 mg) *2‐Escitalopram (10 mg) *3‐Fluoxetine (20 mg/day) *4‐SSRI	*1‐Folic acid (10 mg/per day) *2‐L‐Methylfolate (15 mg) *3‐Folic acid (500 micrograms/day) *4–2.5 mg folic acid *5‐L‐Methylfolate (7.5 mg‐15 mg) *6‐L‐Methylfolate (15 mg)	15 week	Depression ↓	Yes (Cochrane) 3/4 High
Roberts et al. (2018) UK	MDD red‐cell folate below 200 μg/L & MDD	6	NR	NR	*1‐standard psychotropic treatments *2–20 mg fluoxetine *3–20 mg fluoxetine *4‐Any antidepressant at adequate dose and duration *5‐SSRI *6‐SSRI *7‐Citalopram (20 mg/day)	*1–15 mg methyltetrahydrofolate *2–500 μg folic acid *3–10 mg folic acid *4–5 mg *5‐L‐methylfolate (7.5 mg‐15 mg) *6‐L‐methylfolate (15 mg) *7–2.5 mg folic acid	10.87 week	Depression ↓	Yes (GRADE) VERY LOW
Abdullah Al Maruf et al. (2022) Canada	MDD red‐cell folate below 200 μg/L& major depression	3	F/M	44.67	*1‐standard psychotropic treatments *2‐SSRI *3‐SSRI *4‐Escitalopram	*1–15 mg methyltetrahydrofolate *2‐L‐methylfolate (7.5–15 mg) *3‐L‐methylfolate (15 mg) *4‐L‐methylfolate 15 mg	11 week	Depression ↓	Yes (Cochrane) 2/3 High
Altaf et al. (2021) US (Reporting SMD)	NR	5	F/M	40.19	*1‐Fluoxetine (20 mg) *2‐Escitalopram (10 mg) *3‐Fluoxetine (20 mg/day) *4‐Citalopram (20 mg/day) *5‐SSRI *6‐SSRI	*1‐Folic acid (10 mg/per day) *2‐L‐Methylfolate (15 mg) *3‐folic acid (500 micrograms/day) *4–2.5 mg folic acid *5‐L‐Methylfolate (7.5 mg‐15 mg) *6‐L‐Methylfolate (15 mg)	6 week	Depression ↓	Yes (Cochrane) 4/5 High

Also, three meta‐analyses of observational studies, comparing lower‐serum folate levels to higher levels for risk of depression, were included (Gilbody et al., 2007; Jin et al., 2022; Petridou et al., 2016). The included meta‐analyses were conducted between 2007 and 2022. Cross‐sectional, case–control, and cohort studies were included in these meta‐analyses. Characteristics of observational meta‐analyses are shown in Table 2.

**TABLE 2:** Characteristics of meta‐analyses of observational studies investigating the association of folate with depression disorder.

Study, year	Study design	Participants	Study number	Gender	Mean age	Outcome	Quality
Jin et al. (2022)	Observational (cross‐sectional/cohort)	Antenatal depression and postpartum depression	8	F	≤30, >30	A significant relationship between low folate and depression	Yes (Newcastle Ottawa) 7/8 High
Gilbody et al. (2007)	Observational (case–control/cross‐sectional/cohort)	Depression	10	F, M, F/M	50	A significant relationship between low folate and depression	NR
Gilbody et al. (2007)	Observational (cross‐sectional/cohort)	Depression	6	F, M, F/M	50	A significant relationship between low folate and depression	NR
Eleni Th. Petridou et al. (2016)	Observational (case–control/cross‐sectional/cohort)	Depression	11	F, M, F/M	≥55	A significant relationship between low folate and depression	Yes (Newcastle Ottawa) 11/11 High

### 3.3 The effect of folate supplementation on depression based on interventional studies (SMD analyses)

**FIGURE 3:**
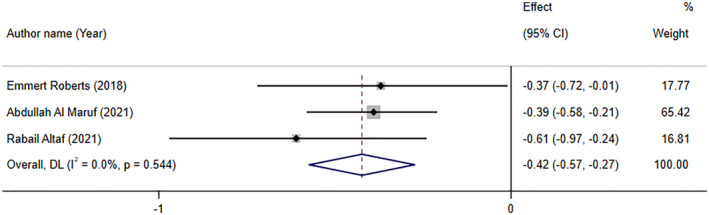
The Forest plot of effects of folate supplementation on depression symptoms based on standardized mean difference (SMD) analyses.

Folate supplementation significantly reduced depression symptoms based on SMD analyses (SMD: −0.42; 95% CI: −0.57, −0.27, *p* < .001), based on the pooled analysis of three meta‐analyses. There was no trace of significant between‐study heterogeneity (*I* ^2^ = 0.0%, p‐heterogeneity = 0.554; Figure 3).

Roberts et al. (2018) performed a meta‐analysis regarding adjunctive folate supplementation in depressive illness, with seven RCTs of 904 participants. Four trials reported investigation of folic acid compared to placebo, two with low dosage (<5 mg/day) and two with high dosage (≥5 mg/day). Three trials reported investigation of methyl folate compared to placebo, two with optimal dose (15 mg/day) and one with a suboptimal dose (<15 mg/day). The overall effect size indicated that depressive symptoms significantly decreased following folic acid supplementation. However, it was of a low quality and considerable heterogeneity. Interestingly, subgroup analysis revealed that there were not any significant changes in depressive symptoms after folate or methyl folate supplementation. Finally, Roberts et al. (2018) recommend not offering either folate or methyl folate as a monotherapy in patients with major depressive disorders. Abdullah Al Maruf et al. (2022) performed a meta‐analysis that included clinical trials evaluating L‐methylfolate supplementation in depressive disorders. There were three studies with four effect sizes in this study. Except for one study, others used 15 mg adjunctive L‐methylfolate. Results revealed that L‐methylfolate supplementation might have modest effect in adults with major depressive disorder (MDD) under treatment with antidepressants. Altaf et al. (2021) evaluated folate as an adjunct therapy to SSRI/SNRI for major depressive disorder with 584 participants. This meta‐analysis, which included five studies with six effect sizes, reported that vitamin B9 significantly improved depression scores evaluated by HAM‐D or BDI‐II. Altaf et al. (2021) reported that participants who were treated with SSRI/SNRI along with folate responded to treatment 36% more than those who were on monotherapy. Furthermore, patients who received folate along with routine medication had a 39% increase in achieving remission in comparison with patients who were on SSRI/SNRI alone (Altaf et al., 2021).

### 3.4 The effect of folate supplementation on depression based on interventional studies (WMD analyses)

**FIGURE 4:**
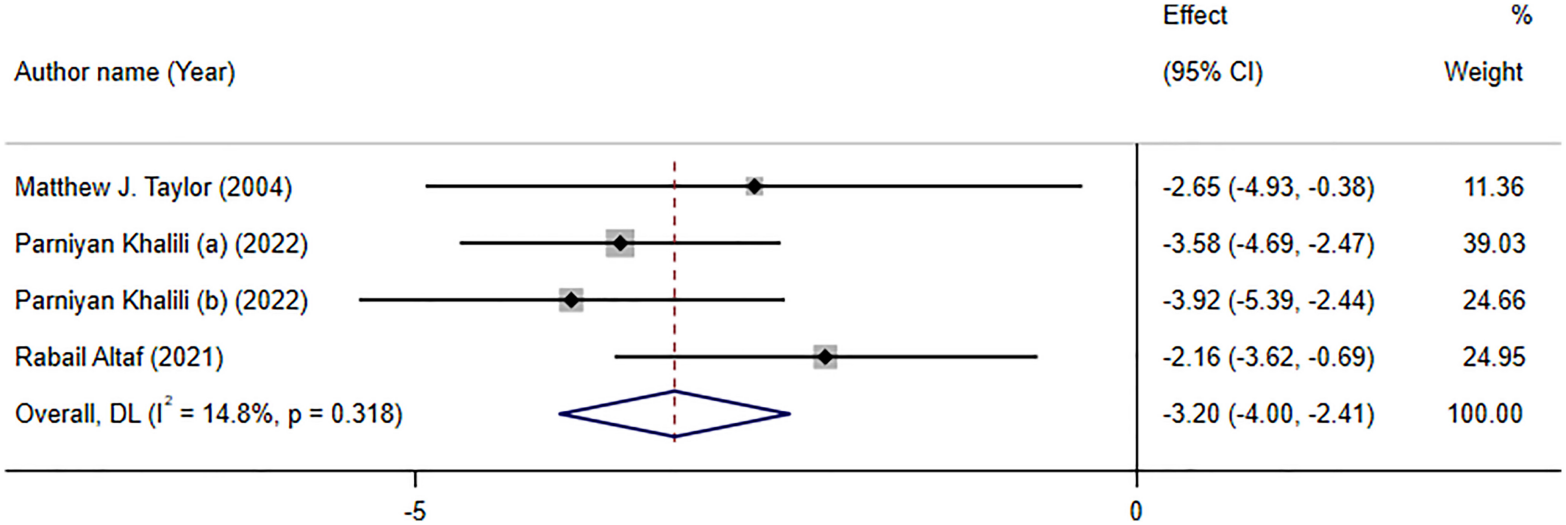
The Forest plot of effects of folate supplementation on depression symptoms based on weighted mean difference (WMD) analyses.

Folate supplementation had a significant impact on relieving depressive symptoms based on WMD analysis (WMD: −3.20; 95% CI: −4.00, −2.41, *p* < .001) of three studies with four effect sizes. No considerable between‐study heterogeneity was found (*I* ^2^ = 14.8%, p‐heterogeneity = 0.318; Figure 4). Subgroup analysis revealed that folate supplementation significantly decreased depression symptoms according to both HAM‐D and BDI scales, with a more robust effect in meta‐analyses with more than 10 studies (See Table 3).

**TABLE 3:** Results of subgroup analyses based on duration of intervention and assessment tool of depression for the effect folate supplementation on depression.

	Effect size *n*	ES (95% CI)	*p*‐within	*I* ^2^ (%)	P‐heterogeneity
Effect of folate supplementation on depression (WMD)					
Overall	3	−3.20 (−4.00, −2.41)	≤.001	14.8	0.318
Duration					
>10	2	−3.54 (−4.78, −2.31)	≤.001	0.0	0.359
≤10	2	−2.95 (−4.34, −1.57)	≤.001	56.4	0.130
Assessment tool					
HAM‐D	3	−2.96 (−3.90, −2.03)	≤.001	16.7	0.301
BDI	1	−3.92 (−5.40, −2.44)	≤.001	–	–

Khalili et al. (2022) performed a meta‐analysis on the effects of folic acid supplementation on depression in adults. This meta‐analysis, with 6 RCTs and a sample size of 308 patients, showed that folate supplementation (0.5–10 mg/day) exerted beneficial impacts on depression test scores, in 6–24 weeks. Taylor et al. (2004) which included only two studies, reported that Hamilton Depression Rating Scale scores were significantly reduced following folate supplementation. One of the included studies used 500 μg/day of folic acid with 20 mg fluoxetine, and the other used 15 mg/day of methyltetrahydrofolate supplementation in addition to the standard psychotropic treatments.

### 3.5 The relationship between low levels of folate and depression based on observational studies

**FIGURE 5:**
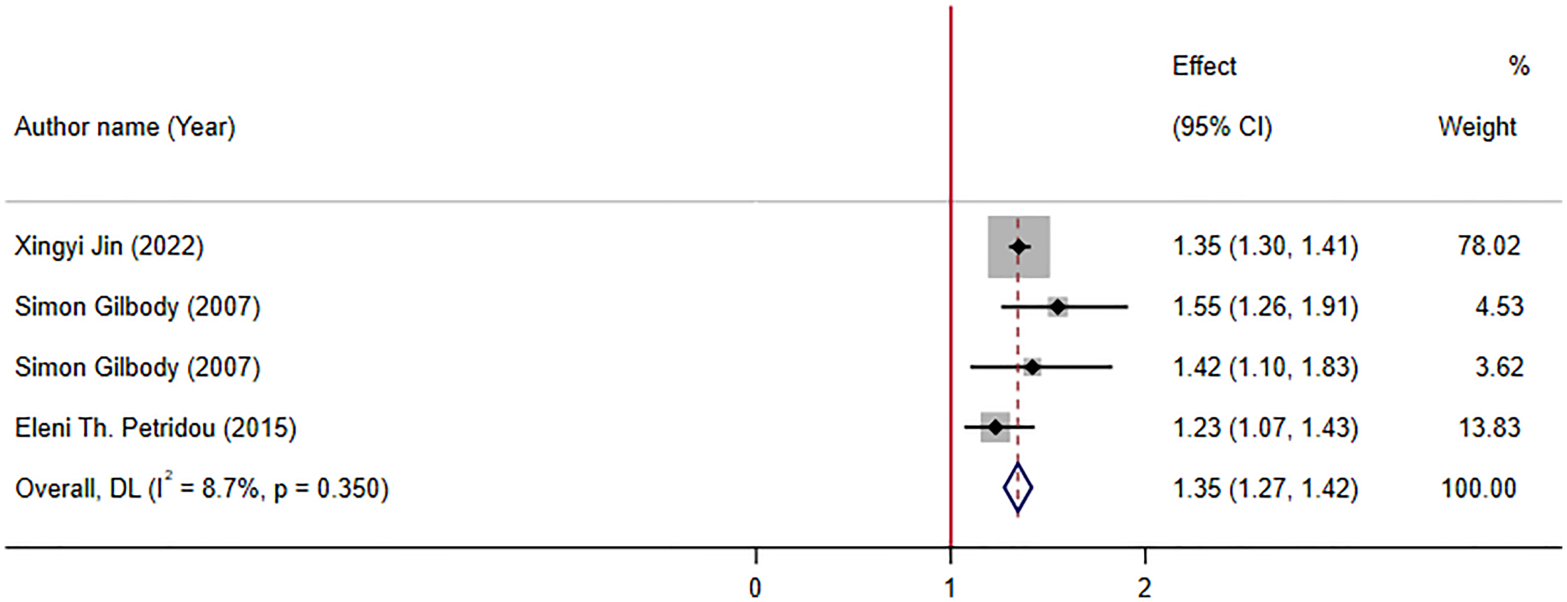
The association low folate serum levels with odds of depression.

In three meta‐analyses of observational studies with four effect sizes, the pooled analysis reported that low levels of folate increase odds of depression by 35% (OR: 1.35; 95% CI: 1.27, 1.42, *p* < .001). There was no significant between‐study heterogeneity (*I* ^2^ = 8.7%, p‐heterogeneity = 0.350) (Figure 5).

Jin et al. (2022) reported that supplementation with folate and folic acid levels are both associated with a reduction in risk of depression symptoms. In another study, Gilbody et al. (2007) included 11 relevant observational studies with 15,315 patients: 3 case–control studies, 7 population surveys, and 1 cohort study. It was reported that there was a significant association between low folate levels and increase in risk of depression. In addition, a meta‐analysis by Petridou et al. (2016) studied the association of folate and vitamin B12 serum levels with depression in the aged population. They reported that “low folate and B12 serum levels are associated with depression in the older individuals.” This meta‐analysis comprised 11 observational studies with 12 effect sizes (9195 participants).

### 3.6 Other related meta‐analyses

There were similar meta‐analyses that were not eligible to be included in the quantitative data synthesis. Ramanujam et al. (2017) pooled correlation coefficient of included data from 758 participants. They reported a significant inverse relationship between folate status and depression. Bender et al. (2017) who reported results as Hedge's g, stated that the serum levels and dietary intake of folate in individuals suffering from depression are lower than in individuals without depression. They suggested that folate supplementation may improve the efficacy of antidepressant medications. However, this study had a high heterogeneity among studies. Therefore, any interpretation should be with caution. Sarris et al. (2016) performed a meta‐analysis on RCTs that evaluated adjunctive nutraceuticals for depression. Four sets of data on folic acid (0.5–10 mg) were included in the meta‐analysis. Two of the four studies revealed a benefit in favor of folic acid; however, the largest study Bedson et al. (2014), with a larger sample size (475 subjects), revealed no significant difference from the placebo. The pooled estimate showed no significant difference between intervention and placebo groups (*p* = .23; *z* = 1.19, 95% confidence interval [CI], −0.31 to 1.29), with an inconsequential effect size (g) of 0.49.

### 3.7 Quality of the studies

According to the AMSTAR2, all included studies were assessed as critically low quality, with items 7 and 4 being the most repeated items that did not meet expected standards. Table 4 highlights the results of quality assessment.

**TABLE 4:** The results of quality assessment included meta‐analyses based on AMSTAR2 questionnaire.

	Q1	Q2	Q3	Q4	Q5	Q6	Q7	Q8	Q9	Q10	Q11	Q12	Q13	Q14	Q15	Q16	Overall
Jin et al. (2022)	Yes	Yes	Yes	Partial Yes	No	Yes	No	Yes	Yes	No	Yes	Yes	Yes	Yes	Yes	Yes	Critically low
Gilbody et al. (2007)	Yes	No	Yes	No	Yes	Yes	No	Yes	No	No	Yes	No	Yes	Yes	Yes	Yes	Critically low
Petridou et al. (2016)	Yes	Yes	Yes	No	Yes	Yes	Yes	Yes	Yes	No	No	No	No	Yes	Yes	Yes	Critically low
Taylor et al. (2004)	Yes	No	Yes	No	Yes	Yes	No	Yes	Partial Yes	No	Yes	No	Yes	Yes	Yes	No	Critically low
Khalili et al. (2022)	Yes	Partial Yes	Yes	Partial Yes	Yes	Yes	No	Yes	Yes	No	Yes	Yes	No	No	Yes	No	Critically low
Altaf et al. (2021)	Yes	Partial Yes	Yes	Yes	No	No	No	Yes	Yes	No	Yes	No	Yes	Yes	Yes	Yes	Critically low
Abdullah Al Maruf et al.	Yes	Yes	Yes	No	Yes	No	No	Yes	Yes	No	Yes	Yes	Yes	Yes	Yes	Yes	Critically low
Roberts et al. (2018)	Yes	Partial Yes	Yes	Yes	Yes	No	No	Yes	Yes	Yes	Yes	No	Yes	Yes	No	Yes	Critically low

*Note*: 1‐ Did the research questions and inclusion criteria for the review include the components of PICO? 2‐ Did the report of the review contain an explicit statement that the review methods were established prior to the conduct of the review, and did the report justify any significant deviations from the protocol? 3‐ Did the review authors explain their selection of the study designs for inclusion in the review? 4‐ Did the review authors use a comprehensive literature search strategy? 5‐ Did the review authors perform study selection in duplicate? 6‐ Did the review authors perform data extraction in duplicate? 7‐ Did the review authors provide a list of excluded studies and justify the exclusions? 8‐ Did the review authors describe the included studies in adequate detail? 9‐ Did the review authors use a satisfactory technique for assessing the risk of bias (RoB) in individual studies that were included in the review? 10‐ Did the review authors report on the sources of funding for the studies included in the review? 11‐ If meta‐analysis was performed, did the review authors use appropriate methods for the statistical combination of results? 12‐ If a meta‐analysis was performed, did the review authors assess the potential impact of RoB in individual studies on the results of the meta‐analysis or other evidence synthesis? 13‐ Did the review authors account for RoB in individual studies when interpreting/discussing the review results? 14‐ Did the review authors provide a satisfactory explanation for and discussion of any heterogeneity observed in the review results? 15‐ If they performed quantitative synthesis, did the review authors conduct an adequate investigation of publication bias (small‐study bias) and discuss its likely impact on the review results? 16‐ Did the review authors report any potential sources of conflict of interest, including any funding they received for conducting the review?.

### 3.8 Sensitivity and publication bias

After performing the sensitivity analysis, the results did not change in all sections. Due to the limited number of observations, we did not perform Egger's and Begg's tests and funnel plots in order to evaluate the small study effect and publication bias.

## 4 DISCUSSION

Although some effective strategies for treating depression have been expanded, remission was not observed in all patients; hence, new treatment strategies focus on adjuvant therapies (Schefft et al., 2017). Previous studies showed increased folate deficiency in patients with depression, suggesting that folate supplements therapy can be a promising treatment strategy (Taylor et al., 2004; Young & Ghadirian, 1989). In this scenario, several meta‐analyses of observational and randomized controlled trial (RCT) studies were conducted to assess the relationship between serum folate levels with depression and whether folate supplements can relieve patients' symptoms; however, conflicting results were obtained (Gilbody et al., 2007; Jin et al., 2022; Khalili et al., 2022; Roberts et al., 2018). The present study is an umbrella review of eight meta‐analyses comprising five meta‐analyses of RCTs and three meta‐analyses of observational studies. This study evaluated how folate supplements as adjuvant therapy can improve depression symptoms and how folate serum levels correlate negatively with depression.

Depression is evaluated by many scales including the Montgomery–Åsberg Depression Rating Scale (MADRS), Profile of Mood States (POMS), Geriatric Depression Scale (GDS), Edinburgh Postnatal Depression Scale (EPDS), Depression Anxiety Stress Scales (DASS), Beck Depression Inventory (BDI), Hamilton Depression Rating Scale (HAM‐D), Leiden Index of Depression (LEID), Quick Inventory of Depressive Symptomatology (QIDS), and Hospital Anxiety and Depression Scale (HADS) (Lee et al., 2010). Each scale is suitable for different functions like disease severity, treatment response, and differentiated diagnosis; for example, BDI is considered the best criterion for moderate depressed patients' severity assessment, while HAM‐D is a gold standard tool for depression evaluation (Carrozzino et al., 2020; Richter et al., 1998).

In the present study, we showed folate supplements as adjuvant therapy can significantly reduce depression symptoms based on the results of both WMD and SMD. Moreover, no significant heterogeneity was observed between meta‐analyses. The results of subgroup analysis indicated folate supplements could enhance depression disorder based on both HAM‐D and BDI criteria; however, their effect was more substantial regarding the BDI criterion. Although we should consider only one effect size assessed BDI, hence the results should be interpreted cautiously. In addition, the results of subgroup analysis regarding treatment duration revealed that both treatment durations with folate supplementation over and below 10 weeks were significantly associated with depression alleviation; however, treatment duration over 10 weeks showed more robust effects.

Although the pooled effect of previous meta‐analyses was significant, there were some controversies among them. Variations in the results can be attributed to different treatment doses and duration, variations in depression scales, different types of analysis, different quality of meta‐analyses, and different sample sizes. For example, in a meta‐analysis of six RCTs, Khalili, Asbaghi, Aghakhani, Clark, & Haghighat (2023) showed that folate supplementation can be promising in depressed patients. Like our study, they revealed that folate supplements are more effective in reducing symptoms based on the BDI than the HAM‐D criterion. They also pointed out that heterogeneity of the analysis was less in BDI compared to HAM‐D, showing more robust results in this regard. Contrary to our study, they showed no significant relationship between duration and dose of treatment with depression symptoms in the HAM‐D criterion. In another meta‐analysis by Altaf et al. (2021) on five studies, folate supplements could significantly improve depression symptoms based on HAM‐D and BDI criteria. Although treatment duration for 4, 6, and 8 weeks significantly affected patients' symptoms, 6‐week treatment had the best response. Another meta‐analysis by Roberts et al. (2018) on seven RCTs showed only low dose of folic acid (<5 mg/day) and methyl folate optimal dose (15 mg/day) had significant effects on alleviating depression symptoms. On the other hand, high dose of folic acid (>5 mg/day) and methyl folate suboptimal dose (<15 mg/day) showed non‐significant results. They reported that their results had low epidemiological strength regarding GRADE (Grading of Recommendations Assessment, Development, and Evaluation) criteria.

Previous evidence from the 1960s suggested that depressed patients have lower‐serum folate levels (Young & Ghadirian, 1989). This fact is not surprising, as depressed patients have low appetites, which results in lower folate absorption. On the other hand, lower folate levels exacerbate depression, and this defective cycle goes on (Young, 2007). Our study demonstrated that low‐serum folate levels could aggravate depression symptoms by up to 35%. The results were accompanied by low heterogeneity. In another meta‐analysis by Bender et al. (2017) depressed patients had lower‐serum folate levels and dietary intake than healthy individuals; however, their finding was accompanied by high heterogeneity. In another meta‐analysis by Petridou et al. (2016), lower folate serum level was significantly associated with depression in females, but a counter‐direction was found in men. The effect of low‐serum folate on other psychological diseases has also been observed. In a meta‐analysis study by Hsieh et al. (2019) 481 bipolar patients were compared with 760 controls and had significantly lower‐serum folate levels. Cao et al. (2016) reported significant serum folate decrease in schizophrenia in a meta‐analysis of 1463 patients and 1276 controls. The results of these studies suggest folate is a crucial factor in brain function, and its deprivation can cause significant health problems.

Among the included observational meta‐analyses, one study did not assess the quality of primary studies (Gilbody et al., 2007). The other two meta‐analyses used the Newcastle Ottawa checklist, and most of their primary studies were considered highly qualified (Jin et al., 2022; Petridou et al., 2016). Five meta‐analyses on RCTs used the Cochrane checklist and rated most of their primary studies as high quality; however, one study used The Grading of Recommendations Assessment, Development, and Evaluation (GRADE) criteria and reported their results as very low epidemiological strength. We assessed the quality of included meta‐analyses in the present study using the AMSTAR2 checklist. This checklist includes 16 questions covering different aspects of meta‐analyses. All the included studies had critically low qualities, indicating that our meta‐umbrella review results may not be conclusive and should be interpreted cautiously; hence, more studies are needed to make our results conclusive. The most prevalent flaws in the included meta‐analyses were as follows: no providing the list of excluded studies and exclusion justification, no assessment regarding the effect of risk of bias of included primary studies on the results, and no identification of the funding source of included primary studies.

Depression is defined as low mood. The present guidelines suggest MDD treatment with monotherapy antidepressants like mirtazapine, bupropion, selective serotonin reuptake inhibitors (SSRIs), and serotonin–norepinephrine reuptake inhibitors (SNRIs) (Clevenger et al., 2018; Mathys & Mitchell, 2011). The mechanism of action of these agents is increasing monoamine transmitters by preventing their reuptake in synapses. Traditional antidepressants have been the foremost choice of depression treatment for years; however, recent studies have shown inadequate response treatment, late response treatment, no response treatment, low efficacy, and significant side effects (Altaf et al., 2021; Blier, 2001; Ginsberg et al., 2011; Jain et al., 2020; Miller, 2008). A novel strategy for increasing the efficacy of depression treatment is the administration of micronutrients like folate (Altaf et al., 2021).

Folate is considered a crucial factor for proper brain and other organ functions (McGarel et al., 2015). Previous studies have shown that folate deficiency may increase depression risk and lower response to antidepressant treatment. There is accumulating evidence that depressed patients who have low‐serum folate levels are more at risk of relapsing and cognitive function worsening (Gilbody et al., 2007; Martínez‐Cengotitabengoa & González‐Pinto, 2017). Supplements with folate can effectively prevent dementia and depression (Mischoulon & Raab, 2007). Animal studies have also shown that folate supplements alone or in combination with other nutrients like omega‐3 fatty acids or zinc induce antidepressant effects (Dou et al., 2018; Gao et al., 2017; Réus et al., 2018).

The exact mechanism of how folate can enhance depression is yet to be understood, but one postulated mechanism is the monoamine hypothesis. Folic acid plays an essential role in synthesizing monoamines like norepinephrine, serotonin, and epinephrine (Rabjohn, 2014). Folic acid cannot be synthesized by the body and is supplied by foods. When entering the body, folic acid converts to its active metabolite, l‐methyl folate. This process is done by a key enzyme called methylene tetrahydrofolate reductase (MTHFR). L‐methyl folate crosses the blood–brain barrier and induces tetrahydrobiopterin (BH4). BH4 is an essential substrate for activating tyrosine hydroxylase and tryptophan hydroxylase, ultimately causing increased production of neurotransmitters like dopamine and 5‐hydroxytryptophan (5‐HTP) (Jain et al., 2020; Rabjohn, 2014). Previous studies illustrated 5‐HT, dopamine, and norepinephrine's crucial role in brain function, cognitive performance, motivation, self‐controlling, and stress reactions (Gu et al., 2018). Moreover, these neurotransmitters are decreased in the hypothalamus of depressed patients (Gu et al., 2019). The fact that folate supplements can increase levels of these neurotransmitters has been confirmed by animal studies (Zhou et al., 2020). Another postulated mechanism for the antidepressant effects of folate is its interaction with IL‐6. Previous studies showed that an increased serum level of IL‐6 is associated with depression (Dowlati et al., 2010; Liu et al., 2012). Moreover, higher levels of IL‐6 can cause treatment resistance in depressed patients (Lanquillon et al., 2000; O'Brien et al., 2007). IL‐6 can decrease BH4, which, as discussed above, is essential for neurotransmitter synthesis. Previous studies reported the anti‐inflammatory effects of folic acid and its ability to reduce IL‐6, resulting in increased neurotransmitter levels (Li et al., 2003; Zhou et al., 2020). Another mechanism of action of folic acid in depressed patients is its effect on homocysteine. Folic acid can reduce homocysteine, a toxic agent for the dopaminergic system (Lee et al., 2005). Previous studies showed that low levels of folic acid and high levels of homocysteine are associated with lower neurotransmitter production in patients with depression (Botez & Young, 1991; Reynolds, 2013). Brain‐derived neurotrophic factor (BDNF) is another substrate folic acid supplements affect. Previous studies indicated that a low level of BDNF is associated with depression, and antidepressant therapy can stimulate BDNF production (Liu et al., 2015). Folic acid can enhance BDNF synthesis resulting in depression healing (Gao et al., 2017; Zhou et al., 2020).

Our study had some advantages. To the best of our knowledge, the present study is the first meta‐umbrella analysis assessing the effect of folate supplements on depression. We assessed the relationship between folate and depression using observational and randomized control trial studies. We also separated the studies with different reporting results units. In addition, we conducted subgroup analysis to determine the effect of treatment duration on depression; however, we had some limitations in this study. The number of included studies was low, and we could not conduct publication bias. We recommend further meta‐analysis studies in this regard. The quality of included primary studies in the meta‐analyses was low and the quality of meta‐analyses was critically low; hence, future highly qualified studies are needed. In addition, we recommend further meta‐analysis to assess folate or methyl folate separately, as these two supplements may act differently. We also suggest further meta‐analyses to assess different treatment doses. None of our included meta‐analyses conducted trial sequential analysis to deal with type 1 and type 2 errors; hence, we suggest such analysis in future studies. As the main population of included meta‐analyses was middle aged, we could not assess the effect of depression on young and old populations; hence, we recommend future studies on these two groups to better understand the effect of age on the folate's effects.

## 5 CONCLUSION

Present umbrella meta‐analysis lends support to using folate supplementation as an efficient adjuvant agent for relieving depression symptoms along with routine medications. The improving effect could be more robust if the supplementation duration lasts for >10 weeks.

## AUTHOR CONTRIBUTIONS

**Shan Gao:** Data curation (lead); investigation (equal); resources (equal). **Awais Khalid:** Data curation (equal); investigation (equal); resources (equal). **Ehsan Amini‐Salehi:** Investigation (equal); writing – original draft (equal). **Nima Radkhah:** Data curation (equal); investigation (equal); writing – original draft (equal). **Parsa Jamilian:** Investigation (equal); writing – original draft (equal). **Mohaddeseh Badpeima:** Investigation (equal); writing – original draft (equal). **Meysam Zarezadeh:** Conceptualization (lead); formal analysis (lead); methodology (lead); project administration (lead); supervision (lead); writing – review and editing (lead).

## FUNDING INFORMATION

This study is supported via funding from Prince Sattam bin Abdulaziz University project number (PSAU/2024/R/1445).

## CONFLICT OF INTEREST STATEMENT

None.

## Data Availability

Data will be made available upon reasonable request.
